# Risk Factors for Ankle Brachial Index and Carotid Artery Stenosis in Patients with Type 2 Diabetes

**DOI:** 10.3390/metabo14010059

**Published:** 2024-01-17

**Authors:** Vesna Đermanović Dobrota, Neva Brkljačić, Angelika Tičinović Ivančić, Maja Čavlović, Tomislav Bulum, Martina Tomić

**Affiliations:** 1 Vuk Vrhovac University Clinic for Diabetes, Endocrinology and Metabolic Diseases, Merkur University Hospital, 10000 Zagreb, Croatia; 2 Zagreb Country Public Health Institute, 10430 Samobor, Croatia; 3 School of Medicine, University of Zagreb, 10000 Zagreb, Croatia

**Keywords:** carotid artery stenosis, atherosclerosis, type 2 diabetes, ankle brachial index

## Abstract

Type 2 diabetes mellitus (T2DM) significantly increases the risk of atherosclerotic cardiovascular disease. Ankle brachial index (ABI) and carotid artery stenosis are non-invasive indicators of generalized atherosclerosis. This study aimed to explore the risk factors for ABI and carotid artery stenosis and discover which factors simultaneously influence both conditions in T2DM. The study included a total of 101 patients with T2DM. ABI was performed via Doppler ultrasound, and both common carotid arteries were examined via ultrasound to obtain the percentage of carotid artery stenosis. A negative correlation was noted between the ABI and the percentage of carotid artery stenosis (*p* = 0.043). ABI correlated significantly negatively with waist circumference (*p* = 0.031), total cholesterol (*p* = 0.003), low-density lipoprotein (LDL) cholesterol (*p* = 0.003), and C-reactive protein (CRP) (*p* = 0.017), whereas the percentage of carotid artery stenosis correlated with the smoking habit (*p* = 0.017) and CRP (*p* = 0.042). The best model for predicting the ABI value (R^2^ = 0.195) obtained from stepwise regression analysis included waist circumference, LDL cholesterol, triglycerides, and CRP, while the best model for the percentage of the carotid artery stenosis (R^2^ = 0.112) included smoking and CRP. CRP influenced the ABI value with a negative parameter estimate of −0.008962 (*p* = 0.053) and the percentage of the carotid artery stenosis with a positive parameter estimate of 0.443655 (*p* = 0.006) relative to a one-unit change of it, presenting the negatively significant impact of CRP on the association between carotid artery stenosis and low ABI. Our results suggest that CRP is the most important risk factor that connects ABI and carotid artery stenosis, which are important non-invasive indicators of generalized atherosclerosis in T2DM.

## 1. Introduction

Diabetes represents an important global health emergency today and is connected with prolific loss of life and premature mortality due to a high incidence of cardiovascular complications [1]. Up to 90% of those with diabetes have type 2 diabetes mellitus (T2DM), and atherosclerotic cardiovascular disorders are the major reason for hospitalization and death in patients with diabetes [2]. Hyperglycemia promotes accelerated atherosclerosis in diabetic patients via several mechanisms, including protein kinase C (PKC) activation, oxidative stress, and the nonenzymatic glycosylation of proteins and lipids [3].

Atherosclerosis is a chronic disorder that affects the arteries of the brain, neck (carotid), heart, aorta, kidneys, and lower extremities. Atherosclerosis is dominant in the medium and large arteries, leading to lipid deposition in tunica intima and media and plaque formation. Advanced inflammation can finally result in the rupture of plaques and acute cardiovascular events [4]. In atherosclerotic plaque, lipid metabolism is closely connected with inflammation, which can additionally stimulate stenosis [5]. Traditionally, atherosclerosis is associated with dyslipidemia, particularly with an elevated level of circulating small dense low-density lipoprotein (LDL) cholesterol, a modified LDL particle that triggers massive lipid accumulation [6].

C-reactive protein (CRP) is an essential factor involved in the initiation and progression of inflammation activating the complement system and promoting phagocytosis. CRP is also useful in diagnosing and stratifying cardiovascular and cerebrovascular risks [7,8]. Atherosclerotic changes and their severity in superficial carotid arteries are usually detected with high-frequency ultrasound, a non-invasive method.

Stroke is the most important condition connected with disability and is placed in second position as a cause of death. About 85–90% of strokes are ischemic, caused by thromboembolism, and around 10–15% of strokes are due to thromboembolism from untreated asymptomatic carotid stenosis [9]. Therefore, screening for carotid artery stenosis in those with multiple vascular risk factors like T2DM is recommended. Even in those with asymptomatic carotid stenosis, drug therapy can reduce the incidence and mortality of cardiovascular and cerebrovascular events and also identify candidates for the surgical treatment of carotid stenosis in a timely manner [10].

Peripheral arterial disease (PAD) and carotid artery stenosis are localized expressions of generalized atherosclerosis caused by the same risk factors with the same underlying pathology. Cerebro- and cardiovascular events more often lead to mortality in patients with PAD [9]. Atherosclerotic changes are simultaneously present and progress in several areas, causing vascular diseases and increasing the probability of cerebrovascular, cardiovascular disease, and PAD [11]. Independently of clinical symptoms, patients with PAD have a significantly higher risk of carotid artery stenosis. Surgical interventions in patients with PAD are more often complicated with stroke, increasing mortality, prolonging hospitalization, and increasing hospitalization costs [12]. Some studies have shown that patients with PAD have a high prevalence of carotid stenosis ranging from 5% to 24%. An ultrasound of the carotid arteries is a proven non-invasive diagnostic test for detecting asymptomatic carotid artery stenosis. Therefore, routine screening before vascular surgery in patients with PAD can be beneficial in preventing the occurrence of stroke [13].

Patients with diabetes have an increased risk of PAD, while patients with PAD have an increased risk of diabetes. The harmful outcomes of diabetes on atherosclerosis and PAD are extended at multiple levels [14]. Although the conventional treatment of PAD is the standardized independently of the presence of diabetes, it should be noted that diabetes significantly increases the risk of cardiovascular disease [15]. A diagnosis of diabetes triples the risk of stroke compared to nondiabetics, and diabetes is closely related to the percentage of carotid artery stenosis [16]. Although diabetes is correlated with an acceptable increased periprocedural risk after stenting of the carotid artery, the diagnosis of diabetes is highly linked to the percentage of carotid artery stenosis and the incidence of in-stent restenosis early after procedures [17]. Besides the plaque size and grade of stenosis, plaque structure is more important in predicting cardiovascular events. Vulnerable plaques are more often in patients with diabetes compared to nondiabetic patients [18]. The preclinical atherosclerosis of the carotid artery as well as carotid artery stenosis is a reflection of localized and systemic polyvascular disorder [19]. In those with significant stenosis of the carotid artery, PAD is present in 21% of patients [20]. The National Institute for Health and Care Excellence (NICE) guidelines and European Society for Cardiology (ECS) guidelines recommend the assessment of PAD presence and its severity by measuring the ankle brachial pressure index (ABI) [21].

T2DM significantly increases the risk of development, progression, and death from atherosclerotic cardiovascular disease. Diabetes is still the leading cause of non-traumatic amputation of the lower extremities. ABI and carotid artery stenosis are non-invasive indicators of generalized atherosclerosis, and it should be essential to identify risk factors that connect cerebral and peripheral artery disease. This study aimed to explore the risk factors for ABI and carotid artery stenosis and discover which factors simultaneously influence both conditions in T2DM.

## 2. Materials and Methods

### 2.1. Study Design, Demographic Data, and Clinical Characteristics

This cross-sectional, single-center (single-hospital) study was performed at the Department of Diabetes and Endocrinology, Department of Neurology, and Department of Cardiology at Merkur University Hospital in Zagreb, Croatia. The second author, N.B., randomly selected and included patients in the study. Since patients with T2DM have a cluster of metabolic syndrome-related disturbances like obesity, dyslipidemia, hypertension, and inflammation risk factors for ABI and carotid artery stenosis included standard demographic data and parameters of obesity, hemoglobin A1c, serum lipids, hypertension, smoking habits, and CRP as a sensitive marker of inflammation. We collected standard demographic data of patients, such as diabetes duration, age, and gender. The parameters of obesity included waist circumference and body mass index (BMI). A digital sphygmomanometer was used to measure systolic and diastolic blood pressure (SBP/DBP) after a 10 min resting period in a sitting posture.

### 2.2. Markers of Glycemic Control and Lipid Metabolism

Laboratory assessment was carried out in the morning on venous blood samples of fasting patients. Standard biochemistry panel in patients with T2DM included fasting glucose, hemoglobin A1c, triglycerides, total cholesterol, high-density lipoprotein (HDL) cholesterol, low-density lipoprotein (LDL) cholesterol, and CRP. Hemoglobin A_1_c was measured using an automated turbidimetric inhibition immunoassay (HbA_1_c Gen 3, Cobas Integra 400 Plus, Roche Diagnostic, Basel, Switzerland) and expressed in National Glycohemoglobin Standardization Program (NGSP) units (%). This method followed the International Federation of Clinical Chemistry and Laboratory Medicine (IFCC) reference system. Standard enzymatic methods were used to measure serum lipids while CRP was measured spectrophotometrically using turbidimetric immuno-inhibition on an automated analyzer (Beckman Coulter AU680, Beckman Coulter, Inc., Brea, CA, USA).

### 2.3. Ankle Brachial Index

To calculate the ABI, the patient remained relaxed in the supine position. The ABI was calculated by measuring the SBP in the brachial, posterior tibial, and dorsalis pedis arteries. The ratio of higher resting SBP at the ankle and higher systolic brachial pressure defines the ABI. We used a handheld 5 or 10 mHz Doppler instrument to measure systolic pressure (Sonotrax Pro ultrasonic pocket Doppler, Edan, San Diego, CA, USA). A standard cuff for blood pressure was placed on the patient’s lower calf above the ankle to obtain the most accurate blood pressure readings. An ABI of <0.9 was considered an abnormal ABI (indicative of peripheral atherosclerosis). Since ABI > 1.3 indicates vascular calcification, those patients were not included in the study [22]. The second author, N.B., a cardiologist, performed ABI result interpretations.

### 2.4. Carotid Ultrasound

A 3–12 MHz linear probe frequency using a Canon Xario 100 G (Canon Medical Systems Corporation, Otawara, Japan) was used to perform the color Doppler ultrasound. The patients were placed in bed in a supine position while their heads were distorted contralaterally by 45° to obtain the most accurate inspection of the common carotid artery (CCA) bifurcation and internal carotid artery (ICA). Examinations of both sides of the ICA and the origin, main part, and bifurcation of the CCA with B-mode imaging of the arterial wall, color Doppler, and spectral Doppler ultrasound were carried out to detect atherosclerosis plaques and to observe blood vessel morphology and endarterium. The spectral Doppler ultrasound of the ICA and CCA was used to record the end-diastolic velocity (EDV) and peak systolic velocity (PSV). The ultrasound examinations were performed by the first author, V.Đ.D., a neurologist with 20 years of experience working with vascular carotid ultrasound. The same neurologist interpreted the results of carotid stenosis. An ICA stenosis of less than 50% was considered not clinically significant, and stenosis greater than 50% was considered clinically significant in T2DM and requires statin therapy as well as regular carotid ultrasound control every 6 to 10 months, depending on the level of the carotid artery stenosis and depending on the hemodynamic speeds of PSV.

### 2.5. Statistical Analysis

Descriptive statistics (means ± SD or median (min–max), and numbers (percentages)) were estimated, including all analyzed continuous and categorical variables (age, gender, diabetes duration, smoking, body mass index (BMI), waist circumference (WC), blood pressure, hemoglobin A1c, serum lipids, CRP, ABI, carotid artery stenosis). In the results, *p* < 0.05 was taken as statistically significant. *t*-tests or Mann–Whitney U and Chi-square tests were used to test the differences between the two ABI groups. In case the assumption of homogeneity of variance was not satisfied, a nonparametric test was used. Correlations between ABI, the percentage of carotid artery stenosis, and all analyzed variables were determined using Spearman’s rank correlation test. Differences in CRP between the groups according to ABI (0.9–1.3, <0.9) and the percentage of carotid artery stenosis (<50%, ≥50%) and their interactions were analyzed with a two-way analysis of variance (ANOVA). The main predictors of ABI and carotid artery stenosis were detected with stepwise multiple linear regression analysis, whose basis is to assess whether only one continuous dependent variable (e.g., in our model, ABI, and in the other model, carotid artery stenosis) can be predicted from a set of independent (or predictor) variables. In other words, a set of predictors explains the variance in one continuous dependent variable. Also, the collinearity in the models was checked, and the collinear variables were removed from the models. All statistical analyses were performed, and the graph was created using the package Statistica™ 14.0.1.

## 3. Results

One hundred and one T2DM were divided according to the ABI into two groups: gr. 1 (ABI = 0.9–1.3) and gr. 2 (ABI < 0.9). The baseline characteristics of the two ABI groups of patients included in the study did not significantly differ in most risk factors like diabetes duration, gender, age, smoking habit, anthropometric parameters BMI and WC, hemoglobin A_1_c, HDL cholesterol, and triglycerides (Table 1). However, patients with an ABI < 0.9 had significantly higher CRP (*p* = 0.018), LDL cholesterol (*p* = 0.017), and total cholesterol (*p* = 0.003) compared to those with ABI = 0.9–1.3. Table 2 presents, similar to Table 1, the baseline features of two groups of patients divided according to carotid artery stenosis. The two groups had similar age, gender, diabetes duration, anthropometric parameters, BMI, WC, hemoglobin A1c, total, HDL and LDL cholesterol, and triglycerides data. However, patients with carotid artery stenosis ≥ 50% had significantly more frequent smoking habits (*p* = 0.030) and marginally higher CRP (*p* = 0.064) than those with carotid artery stenosis < 50%. Those with ABI < 0.9 had a higher percent of carotid artery stenosis (*p* = 0.005) than those with ABI = 0.9–1.3 (Table 1), and those with clinically significant stenosis ≥ 50% had significantly lower ABI (*p* = 0.020) than those with carotid artery stenosis < 50% (Table 2).

Since the measurements of ABI and the carotid artery stenosis between two sides (right and left) within the two ABI groups were similar without significant differences, the mean values of ABI and carotid artery stenosis of both sides were used in further statistical analyses.

ABI and the percentage of carotid artery stenosis correlated significantly negatively (*p* = 0.043) (Table 3). ABI also correlated significantly negatively with CRP (*p* = 0.017), WC (*p* = 0.031), total cholesterol (*p* = 0.003), and LDL cholesterol (*p* = 0.003), whereas the percentage of carotid artery stenosis related significantly positively to smoking habit (*p* = 0.017) and CRP (*p* = 0.042). The table does not show the nonsignificant relationships of ABI, the percentage of carotid artery stenosis, and other analyzed variables.

**Figure 1:**
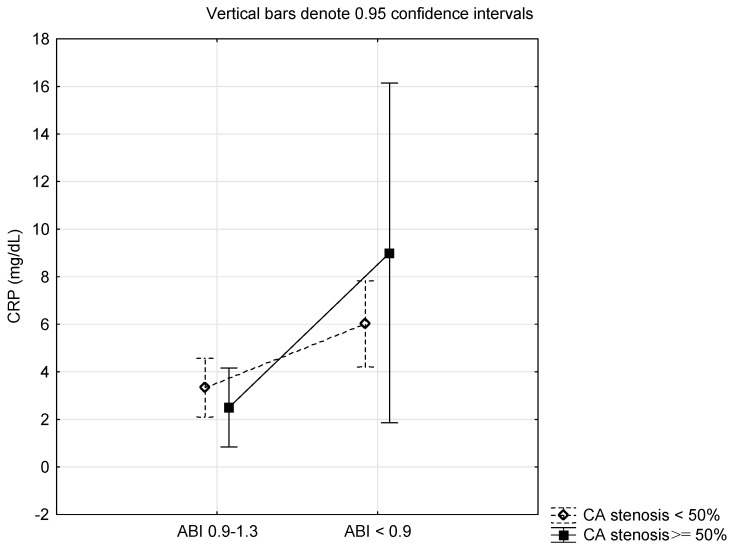
Differences in C-reactive protein according to the ankle brachial pressure index (ABI) and carotid artery (CA) stenosis.

Table 4 presents the differences in CRP of T2DM stratified into two groups according to the level of ABI and carotid artery stenosis tested using ANOVA with two main factors and their interactions. CRP level was statistically significantly different concerning the level of ABI (*p* = 0.0019). However, CRP level was not statistically significantly different concerning the degree of carotid artery stenosis and in the interaction between the groups of ABI and carotid artery stenosis (*p* > 0.05). Differences in CRP level observed via ANOVA according to the ABI and carotid artery stenosis are present in Figure 1.

The best model for predicting the ABI value (R^2^ = 0.195) obtained from stepwise regression analysis included WC, LDL cholesterol, triglycerides, and CRP (Table 5), while the best model for the degree of the carotid artery stenosis (R^2^ = 0.112) included smoking and CRP (Table 6). CRP influenced the ABI value with a negative parameter estimate of −0.008962 (*p* = 0.053) and the percentage of the carotid artery stenosis with a positive parameter estimate of 0.443655 (*p* = 0.006) relative to a one-unit change of it, presenting the negatively significant impact of CRP on the association between carotid artery stenosis and low ABI.

## 4. Discussion

The results of our study suggest that CRP might be an important risk factor that connects ABI and carotid artery stenosis, which are non-invasive indicators of generalized atherosclerosis in T2DM. T2DM is one of the most prominent burdens in clinical medicine, affecting millions worldwide. T2DM causes significant microvascular and macrovascular diseases leading to a higher risk of cardiovascular death [23]. The immune system plays a significant role in developing atherosclerosis, which is accelerated in T2DM. A well-known sensitive marker of innate immune system activation and inflammation is CRP. CRP is a short pentraxine secreted by the liver and belonging to a group of acute phase proteins in response to Interleukin-6, released from M1 macrophages stimulated by microbial molecular pattern ligands or danger-associated molecular pattern ligands [24]. Several studies connected elevated concentrations of CRP with atherosclerosis, such as coronary heart disease and stroke [25]. Those with multivessel coronary disease have the highest serum level of CRP. It was also found that serum CRP concentration and carotid plaque structure closely correlate with the severity of cardiovascular disease [26]. High levels of CRP stimulate the expression of chemokines and adhesion molecules and promote inflammation.

Atherosclerosis has a complex pathogenesis. Atherosclerosis causes lipid infiltration and the injury of endothelial cells, while macrophages are transformed into foam cells loaded with lipids [27]. The essential underlying condition in atherosclerosis’s onset, development, and progression is an inflammatory response. The disease’s severity can correspond with inflammatory marker levels [28]. CRP has a significant role in the pathogenesis of the development and progression of atherosclerosis. An increased level of serum CRP is now considered a marker and predictor of the severity of cardiovascular disease and events. To a certain extent, the higher percentage of carotid artery stenosis is indicative of advanced coronary disease. Plaques on the carotid artery reflect advanced atherosclerosis caused by endothelial dysfunction, oxidation, and smooth muscle cell proliferation [26,29,30].

Patients with PAD often have other risk factors for atherosclerosis and have a higher prevalence of carotid artery stenosis compared to the general population. In people over 70 years old, an ABI of less than 0.5 is a vital risk factor for asymptomatic carotid artery stenosis in those with PAD [31]. However, in that study, diabetes, hypercholesterolemia, hypertension, and smoking were not risk factors for asymptomatic carotid artery stenosis, indicating that these are risk factors for systemic atherosclerosis. Advanced age, along with hypercholesterolemia, were found to be significant predictors of carotid atherosclerosis in PAD, and age over 65 years is significantly associated with carotid artery stenosis above 70% [32]. Those with asymptomatic carotid artery stenosis have to strictly control risk factors to decrease the incidence of cardiovascular diseases. Those with lower ABI also have an increased risk of other systemic atherosclerotic diseases like cardiovascular disease, transient ischemic attack, and stroke [33]. In the Japanese population, an ABI below 0.9 significantly increases the risk of stroke and asymptomatic carotid artery stenosis [34]. A low ABI is also a predictor of asymptomatic cerebrovascular disease in patients with PAD requiring intervention.

In our study, we did not deal with the characteristics of the carotid plaque because plaques with dominantly necrotic core, hemorrhage into the plaque, or damage to the lumen surface are highly associated with cerebrovascular events in asymptomatic or symptomatic stenosis of carotid arteries 30–99% [34]. An ABI of less than 0.7 is associated with significant carotid artery stenosis, i.e., above 50%, while an ABI less than 0.3 and multiple vascular lesions of iliac vessels are essential factors affecting the percent of internal carotid artery stenosis in PAD [13]. Carotid stenosis above 70% or unstable plaque indicates that age over 65 years, cardiovascular disease, and chronic kidney disease are significant predictors for asymptomatic carotid stenosis in PAD.

CRP level relates to the level of carotid artery stenosis [35]. The direct pathogenic role of CRP in the process of atherosclerosis and plaque formation is well-known even in women who are healthy but have a history of smoking [36,37,38,39]. The inflammatory process is elevated in smokers and linked to the length of years of smoking [40]. Obesity increases systemic inflammation and leads to atherosclerosis. A high correlation between subclinical atherosclerosis and hemoglobin A1c and the duration of diabetes affects plaques and the stenosis of coronary arteries, the atherosclerosis of carotid arteries, and ABI [41]. The progression of atherosclerosis in the carotid and coronary arteries is aggravated by hyperglycemia [42]. In addition, the decline in ABI is more substantial in poorly controlled patients with diabetes [43]. Subclinical atherosclerosis and plaque in carotid and coronary arteries are more common in diabetes and prediabetes, and over 50% of asymptomatic T2DM have subclinical atherosclerosis [44].

Besides CRP, novel biomarkers like matrix metalloproteinases (MMPs) 3,7, and 9 and the tissue inhibitors of metalloproteinase (TIMP)-1 are elevated in those with carotid atherosclerosis, and the elevation of those biomarkers parallel with the percentage of carotid artery stenosis. Patients with elevated TIMP-1, MMP-3, MMP-7, and MMP-9 more often require carotid revascularization than those without carotid artery stenosis or those under conservative treatment [45]. The early detection of the inflammation and calcification at the molecular level in bilateral carotid artery atherosclerosis was recently detected using positron emission tomography/computed tomography (PET/CT) with (18F) sodium fluoride (NaF) as a tracer. In that study, (18F) NaF uptake significantly correlated with age, male gender, obesity, fibrinogen, and CRP compared to healthy controls [46].

Our study has several limitations. First, this was a single hospital-based study that included a small number of T2DM patients. Therefore, selection bias is likely, and the ability to generalize and reliably replicate the results is limited. Second, a single physician performed the examinations of carotid stenosis without independent control from another one. Third, this cohort included a white European population without racial/ethnic diversity. Fourth, the design of the study was cross-sectional, which limited the temporal relationship between potential risk factors and outcomes.

## 5. Conclusions

It is most important to diagnose PAD and carotid artery stenosis in the early stage of the disease to improve the quality of life and prevent death from cardiovascular diseases and non-traumatic amputation of the lower extremities. T2DM induces the development of atherosclerosis and further accelerates its progression. Several risk factors are associated with atherosclerosis in T2DM. Our results suggest that CRP might be an important risk factor that connects ABI and carotid artery stenosis, which are non-invasive indicators of generalized atherosclerosis in T2DM. Patients with T2DM and increased CRP should be screened for PAD and carotid artery stenosis.

## Figures and Tables

**Table 1:** Baseline characteristics of type 2 diabetic patients (*n* = 101) divided into two groups according to the ankle brachial index.

	ABI 0.9–1.3 (*n* = 28)	ABI < 0.9 (*n* = 73)	t^a^ χ^b^ Z^c^	*p*-Value
Age (years)	67.11 ± 7.24	68.78 ± 8.37	−1.309 ^a^	0.192
Gender (m/f) (%)	72.7/27.3	73.5/26.5	0.113 ^b^	0.915
Diabetes duration (years)	19 (1–37)	19 (1–44)	−0.704 ^c^	0.481
Smoking (no/yes) (%)	52.7/47.3	47.6/52.4	0.418 ^b^	0.518
BMI (kg/m^2^)	29.24 ± 5.18	30.24 ± 4.96	−1.242 ^a^	0.216
WC (cm)	105.66 ± 13.67	109.35 ± 12.28	−1.489 ^a^	0.139
SBP (mmHg)	140 (100–210)	140 (90–235)	−0.279 ^c^	0.779
DBP (mmHg)	80 (41–110)	80 (60–115)	−0.795 ^c^	0.427
HbA_1_c (%)	7.87 ± 2.03	8.10 ± 1.51	−0.886 ^c^	0.377
Total cholesterol (mmol/L)	4.1 (2.5–7.3)	4.8 (2.9–12.2)	−2.951 ^c^	0.003
HDL cholesterol (mmol/L)	1.2 (0.6–2.2)	1.2 (0.6–1.8)	0.749 ^c^	0.454
LDL cholesterol (mmol/L)	2.2 (0.3–4.7)	2.5 (1.2–8.0)	−2.386 ^c^	0.017
TG (mmol/L)	1.5 (0.8–5.4)	1.7 (0.6–24.4)	−1.314 ^c^	0.189
CRP (mg/dL)	3.04 ± 2.32	6.19 ± 3.01	−2.366 ^a^	0.018
ABI	1.0 (0.9–1.3)	0.7 (0.3–0.89)	10.929 ^c^	<0.001
Carotid artery stenosis (%)	37 (15–55)	41 (20–70)	−2.822 ^c^	0.005

Legend: Values are means ± SD, percentages, or medians (min–max). t^a^: represents *t*-test; χ^b^: chi-square test; Z^c^: Mann–Whitney test; *p*-values comparison between patients with different levels of ABI. ABI indicates ankle brachial index; BMI: body mass index; WC: waist circumference; SBP: systolic blood pressure; DBP: diastolic blood pressure; HbA_1_c: hemoglobin A1c; HDL: high-density lipoprotein cholesterol; LDL: low-density lipoprotein cholesterol; TG: triglycerides; CRP: C-reactive protein.

**Table 2:** Baseline characteristics of type 2 diabetic patients (*n* = 101) divided into two groups according to the carotid artery stenosis.

	CAS < 50% (*n* = 78)	CAS ≥ 50% (*n* = 23)	t^a^ χ^b^ Z^c^	*p*-Value
Age (years)	68.16 ± 7.99	69.30 ± 8.48	−0.815 ^a^	0.416
Gender (m/f) (%)	72.6/27.4	74.4/25.6	0.056 ^b^	0.813
Diabetes duration (years)	19 (1–44)	18 (3–34)	1.079 ^c^	0.280
Smoking (no/yes) (%)	53.5/46.5	34.9/65.1	4.681 ^b^	0.030
BMI (kg/m^2^)	29.32 ± 4.85	29.74 ± 5.81	−0.467 ^a^	0.641
WC (cm)	106.70 ± 12.97	105.83 ± 15.69	0.387 ^a^	0.699
SBP (mmHg)	140 (90–235)	140 (90–180)	0.706 ^c^	0.480
DBP (mmHg)	80 (41–110)	80 (60–115)	1.113 ^c^	0.258
HbA_1_c (%)	8.06 ± 1.76	7.92 ± 1.51	0.474 ^c^	0.636
Total cholesterol (mmol/L)	4.5 (2.5–9.2)	4.4 (3.0–9.2)	0.483 ^c^	629
HDL cholesterol (mmol/L)	1.2 (0.6–2.3)	1.2 (0.8–2.2)	0.343 ^c^	0.731
LDL cholesterol (mmol/L)	2.3 (0.3–4.9)	2.6 (1.3–4.9)	0.189 ^c^	0.850
TG (mmol/L)	1.6 (0.6–9.5)	1.6 (0.7–24.4)	0.874 ^c^	0.382
CRP (mg/dL)	2.83 ± 2.71	4.05 ± 5.80	−1.621 ^a^	0.064
Carotid artery stenosis (%)	40 (10–45)	50 (50–70)	−10.037 ^c^	<0.001
ABI	0.8 (0.3–1.3)	0.7 (0.5–1.2)	2.322 ^c^	0.020

Legend: Values are means ± SD, percentages, or medians (min–max). t^a^: represents *t*-test; χ^b^: chi-square test; Z^c^: Mann–Whitney test; *p*-values comparison between patients with different levels of CAS. CAS indicates carotid artery stenosis; BMI: body mass index; SBP: systolic blood pressure; DBP: diastolic blood pressure; HbA_1_c: hemoglobin A1c; HDL: high-density lipoprotein cholesterol; LDL: low-density lipoprotein cholesterol; TG: triglycerides; CRP: C-reactive protein; ABI: ankle brachial index.

**Table 3:** Correlation between ABI and percent of carotid artery stenosis, as well as their relationships to smoking, waist circumference, total and LDL cholesterol, and C-reactive protein.

	ABI			Percentage of CA Stenosis		
	Spearman’s R	t(N-2)	*p*-Value	Spearman’s R	t(N-2)	*p*-Value
Percent of CA stenosis	−0.143	−2.044	0.043		/	
Smoking	−0.017	−0.235	0.814	0.167	2.401	0.017
WC	−0.180	−2.184	0.031	0.117	1.407	0.162
Total cholesterol	−0.206	−2.973	0.003	0.059	0.836	0.404
LDL cholesterol	−0.212	−3.061	0.003	0.050	0.710	0.478
CRP	−0.203	−2.412	0.017	0.174	2.054	0.042

Legend: ABI: indicates ankle brachial index; CA: carotid artery; WC: waist circumference; LDL: low-density lipoprotein cholesterol; CRP: C-reactive protein.

**Table 4:** Results of two-way ANOVA for the differences between C-reactive protein according to the ABI, carotid artery stenosis, and their interaction.

		CRP	
	df	F	*p*
ABI	1	5.616	0.019
CA stenosis	1	0.309	0.579
ABI and CA stenosis	1	0.972	0.326

Legend: CRP: indicates C-reactive protein; ABI: ankle brachial index; CA: carotid artery.

**Table 5:** Results of stepwise regression analysis for ABI as a dependent variable.

Variable	Estimate	−95%CI	+95%CI	Standard Error	F	*p*-Value	Adjusted R^2^	R^2^
WC	−0.003	−0.005	−0.001	0.201	4.83	0.0295	0.0261	0.195
LDL cholesterol	−0.037	−0.063	−0.010	0.192	7.49	0.006	0.0316	
Triglycerides	−0.014	−0.034	−0.006	0.195	3.47	0.0497	0.0438	
CRP	−0.009	−0.015	−0.002	0.194	8.31	0.0046	0.0517	

Legend: CI: indicates confidence interval of estimate; ABI: indicates ankle brachial index; WC: waist circumference; LDL: low-density lipoprotein cholesterol; CRP: C-reactive protein.

**Table 6:** Results of stepwise regression analysis for the percentage of carotid artery stenosis as a dependent variable.

Variable	Estimate	−95%CI	+95%CI	Standard Error	F	*p*-Value	Adjusted R^2^	R^2^
Smoking	1.761	0.181	3.174	10.723	5.44	0.0206	0.0216	0.112
CRP	0.444	0.135	0.767	9.789	7.71	0.0060	0.0470	

Legend: CI: indicates confidence interval of estimate; CRP: indicates C-reactive protein.

## Data Availability

The data presented in this study are available on a specific request from the corresponding author. The data are not publicly available due to privacy.
